# Anthroponotic transmission of *Cryptosporidium parvum* predominates in countries with poorer sanitation: a systematic review and meta-analysis

**DOI:** 10.1186/s13071-018-3263-0

**Published:** 2019-01-08

**Authors:** Philippa King, Kevin M. Tyler, Paul R. Hunter

**Affiliations:** 1 The Norwich Medical School, University of East Anglia, Norwich, UK; 2 Department of Environmental Health, Tshwane University of Technology, Pretoria, South Africa

**Keywords:** *Cryptosporidium parvum*, Subtypes, Epidemiology, Sanitation

## Abstract

**Background:**

Globally cryptosporidiosis is one of the commonest causes of mortality in children under 24 months old and may be associated with important longterm health effects. Whilst most strains of *Cryptosporidium parvum* are zoonotic, *C. parvum* IIc is almost certainly anthroponotic. The global distribution of this potentially important emerging infection is not clear.

**Methods:**

We conducted a systematic review of papers identifying the subtype distribution of *C. parvum* infections globally. We searched PubMed and Scopus using the following key terms Cryptospor* AND parvum AND (genotyp* OR subtyp* OR *gp60*). Studies were eligible for inclusion if they had found *C. parvum* within their human study population and had subtyped some or all of these samples using standard *gp60* subtyping. Pooled analyses of the proportion of strains being of the IIc subtype were determined using StatsDirect. Meta-regression analyses were run to determine any association between the relative prevalence of IIc and Gross Domestic Product, proportion of the population with access to improved drinking water and improved sanitation.

**Results:**

From an initial 843 studies, 85 were included in further analysis. *Cryptosporidium parvum* IIc was found in 43 of these 85 studies. Across all studies the pooled estimate of relative prevalence of IIc was 19.0% (95% CI: 12.9–25.9%), but there was substantial heterogeneity. In a meta-regression analysis, the relative proportion of all *C. parvum* infections being IIc decreased as the percentage of the population with access to improved sanitation increased and was some 3.4 times higher in those studies focussing on HIV-positive indivduals.

**Conclusions:**

The anthroponotic *C. parvum* IIc predominates primarily in lower-income countries with poor sanitation and in HIV-positive individuals. Given the apparent enhanced post-infectious virulence of the other main anthroponotic species of *Cryptosporidium* (*C. hominis*), it is important to learn about the impact of this subtype on human health.

**Electronic supplementary material:**

The online version of this article (10.1186/s13071-018-3263-0) contains supplementary material, which is available to authorized users.

## Background

*Cryptosporidium* spp. are enteric protozoan parasites found globally throughout the world. They are ubiquitous in the environment and capable of causing infection in both immunocompetent and immunocompromised humans as well as a wide variety of animals. Their ability to cause both sporadic episodes of disease as well as more far-reaching food and waterborne outbreaks is becoming increasingly recognised [1, 2]

*Cryptosporidium* spp. are now one of the major causes of mortality from an infectious disease in children under 24 months in low income countries and are associated with an increased risk of death in toddlers aged 12–23 months [3]. Even in children who survive, there is growing evidence of a link between cryptosporidiosis, childhood malnutrition and stunting in such countries [4]. It has been estimated that in 2015 1.3 million deaths worldwide were due to diarrhoeal disease, and *Cryptosporidium* spp. are the second commonest cause of death from diarrhoeal disease in children under five years, after Rotavirus [5]. However, whilst Rotavirus has an internationally available vaccine that is successfully reducing numbers of severe gastro-intestinal disease and death [6], *Cryptosporidium* spp. have neither a vaccine nor effective treatment to reduce its morbidity and mortality.

Although there have been reported cases of human infection from at least 17 species of *Cryptosporidium*, *C. hominis* and *C. parvum* are the two species that have been most associated with causing human disease [7].

Subtyping of *Cryptosporidium* species, specifically *C. hominis* and *C. parvum*, can provide clarity of mode of transmission in addition to being important epidemiological tools, especially in outbreak situations. At present, the most commonly used and accepted method of subtyping is through sequencing of the *gp60* gene, a 60-kDa glycoprotein. The *gp60* gene has a number of tandem repeats at the 5' end of the gene consisting of TCA, TCG or TCT [8] and also further variation in the non-repeat 3' end of the gene, enabling classification of *C. hominis* and *C. parvum* into subtype families on the basis of the number and type of trinucleotide repeat [9].

*Cryptosporidium parvum* IIc is of particular interest as it appears to be anthroponotic, i.e. host- restricted to humans, which is in direct contrast to most other subtype families of *C. parvum*, which cause disease in both humans and animals [8].

*Cryptosporidium* genotypes and isolates vary in their virulence, with over 25 putative virulence factors identified [10]. Hunter et al. [11] showed that *C. hominis* was associated with an increased risk of post-infectious sequelae compared with *C. parvum* [11] and Cama et al. [12] suggested *C. hominis* Ib is more pathogenic than other subtypes. However, the differences in virulence of *C. parvum* subtypes have not been systemically studied, and there are very little data available linking subtype to pathology. As existing evidence suggests anthroponotic *Cryptosporidium* spp. are more virulent than zoonotic *Cryptosporidium* spp, then as *C. parvum* IIc is transmitted anthroponotically like *C. hominis*, it may display enhanced virulence like *C. hominis*.

The worldwide distribution of *C. parvum* IIc has not been systematically studied or documented. Thus we aimed to identify all studies that had subtyped *C. parvum* using *gp60* subtyping methods, to characterise the endemic worldwide distribution and proportion of *C. parvum* IIc and investigate how this differs throughout the world, in particular exploring potential links with gross domestic product (GDP), a measure of economic growth and often used as a proxy for standard of living, and sanitation, in order to provide clarity on proportion and distribution of anthroponotic *C. parvum* IIc.

## Methods

### Search strategy

PubMed and Scopus were searched up to and including 1st November 2016 using the following search strategy: Cryptospor* AND parvum AND (genotyp* OR subtyp* OR *gp60*) without restriction on language or study type. Review articles and book chapters were additionally reviewed for references which would fit the inclusion criteria.

### Eligibility criteria

Studies were eligible for inclusion if they had found *C. parvum* within their study population and had subtyped some or all of these samples using the standard *gp60* subtyping classification. The study population of interest was restricted to humans, thus studies which had subtyped *C. parvum* found in animals were excluded, but for studies that had included both animals and humans the subtyping data for humans only was included. Reviews and editorials were excluded. Studies which reported subtyping of outbreaks were excluded as the aim was to identify the endemic worldwide proportion of *C. parvum* IIc.

If studies had included data from a previous study, only one study was included to prevent duplication of data.

Data including country of study, population characteristics, subtypes found, number of samples of subtypes found and total number of *C. parvum* samples subtyped were extracted onto a datasheet by one of the researchers. Independently a second researcher reviewed the studies included in the search and their inclusion or exclusion in the final dataset.

Eligible studies were screened initially by title and abstract, and included if they met the inclusion criteria and full text retrieved. If it was unclear from the title and abstract whether the inclusion criteria were met, then the full paper was reviewed and decision made regarding inclusion or exclusion.

### Statistical analysis

Proportion of *C. parvum* IIc in relation to total *C. parvum* was calculated using the number of samples of *C. parvum* IIc and the total *C. parvum* samples subtyped using *gp60* subtyping classification. Forest plots and pooled prevalence estimations were performed using STATS Direct™. When investigating drivers of heterogeneity, negative binomial regression analyses were carried out using STATA™. Given the known association of cryptosporidiosis with drinking water associated outbreaks, the faecal oral transmission pathway and the anthroponotic nature of *C. parvum* IIc, we hypothesised that much of the heterogeneity between countries could be explained by variation in access to improved drinking water and sanitation. In constructing the regression analyses we took data on Gross Domestic capita per person, % of the population with access to improved sanitation and % of the population with access to an improved water supply. All three variables were taken from the World Bank World Development Indicators Archive [13] and for the year when the study was undertaken or published. Gross domestic product per capita (GDPpc) was expressed in USD for year 2005. The definitions of improved sanitation and improved water supply were as used by the World Bank which are themselves taken from the definitions of the WHO/UNICEF Joint Monitoring Programme [14]. All three country-specific variables were taken to be two years prior to the publication date to adjust for time of collection to publication. Given the marked skewedness of the GDP data we used the log_10_-transformed data. In addition, publication year and whether or not a focus of the study was C*ryptosporidium* infections in HIV-positive individuals were also included in the analysis. The regression analysis was run with all predictors individually and then all in a single model removing the least statistically significant until all variables were significant at the *P* < 0.2 level.

PRISMA guidelines were followed in the preparation of this manuscript (see PRISMA checklist in Additional file 1: Table S1).

## Results

The PubMed search gave a total of 750 results, and the Scopus search revealed an additional 90 unique studies. Reviewing of references in review articles and book chapters identified three further studies with subtyping information on *C. parvum*, resulting in a total of 843 unique studies.

**Fig. 1:**
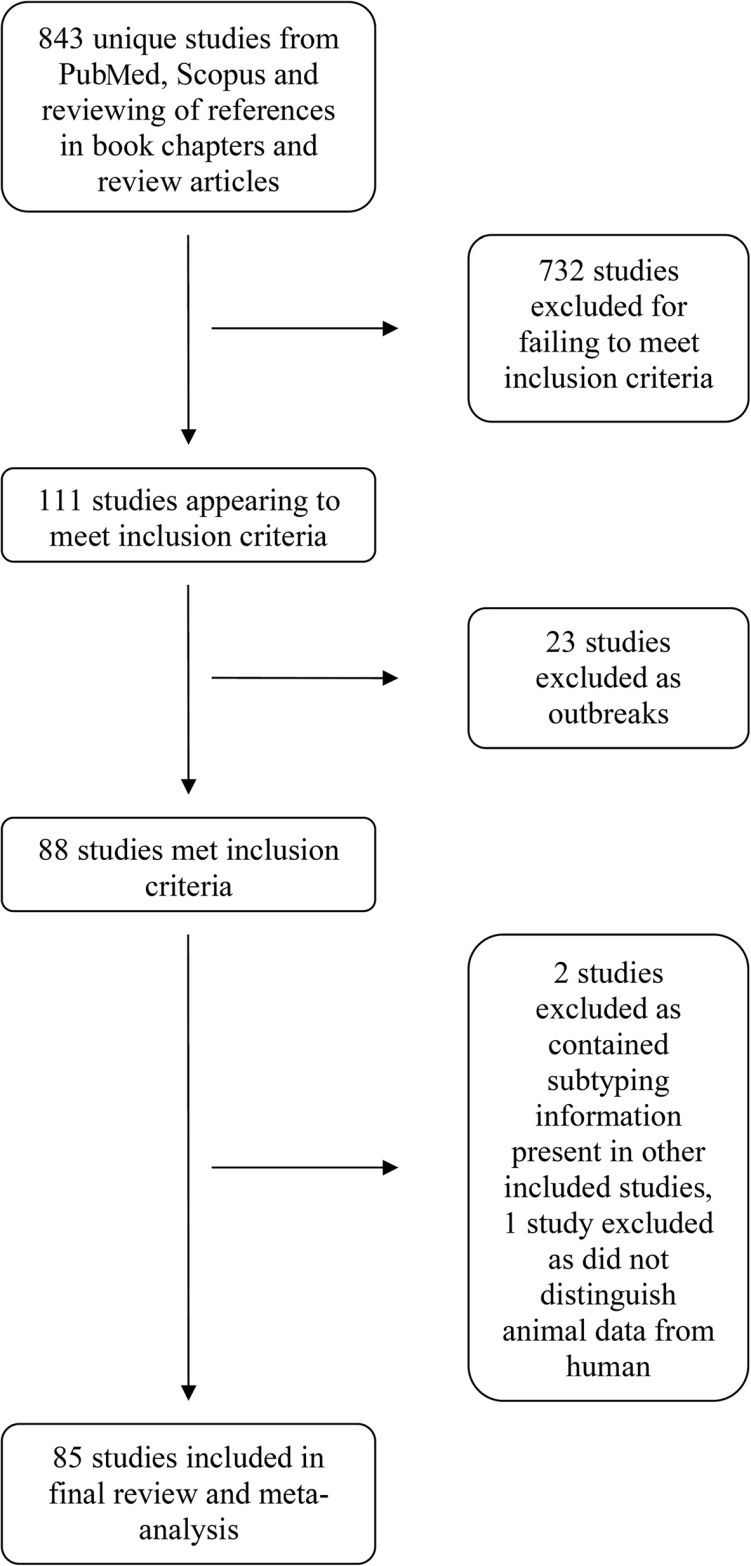
Flow chart depicting inclusion and exclusion of studies with numbers

A total of 732 studies were excluded for failing to meet the inclusion criteria, mainly because they were not original studies with subtyping information or had only included animals or environmental samples. A further 23 studies were excluded as they were studies of outbreaks, as the aim was to characterise the endemic proportion of *C. parvum* IIc. Two further studies were excluded as they contained duplication of material presented in other already included studies. The final number of studies included was 85 (Fig. 1, Table 1).

**Fig. 2:**
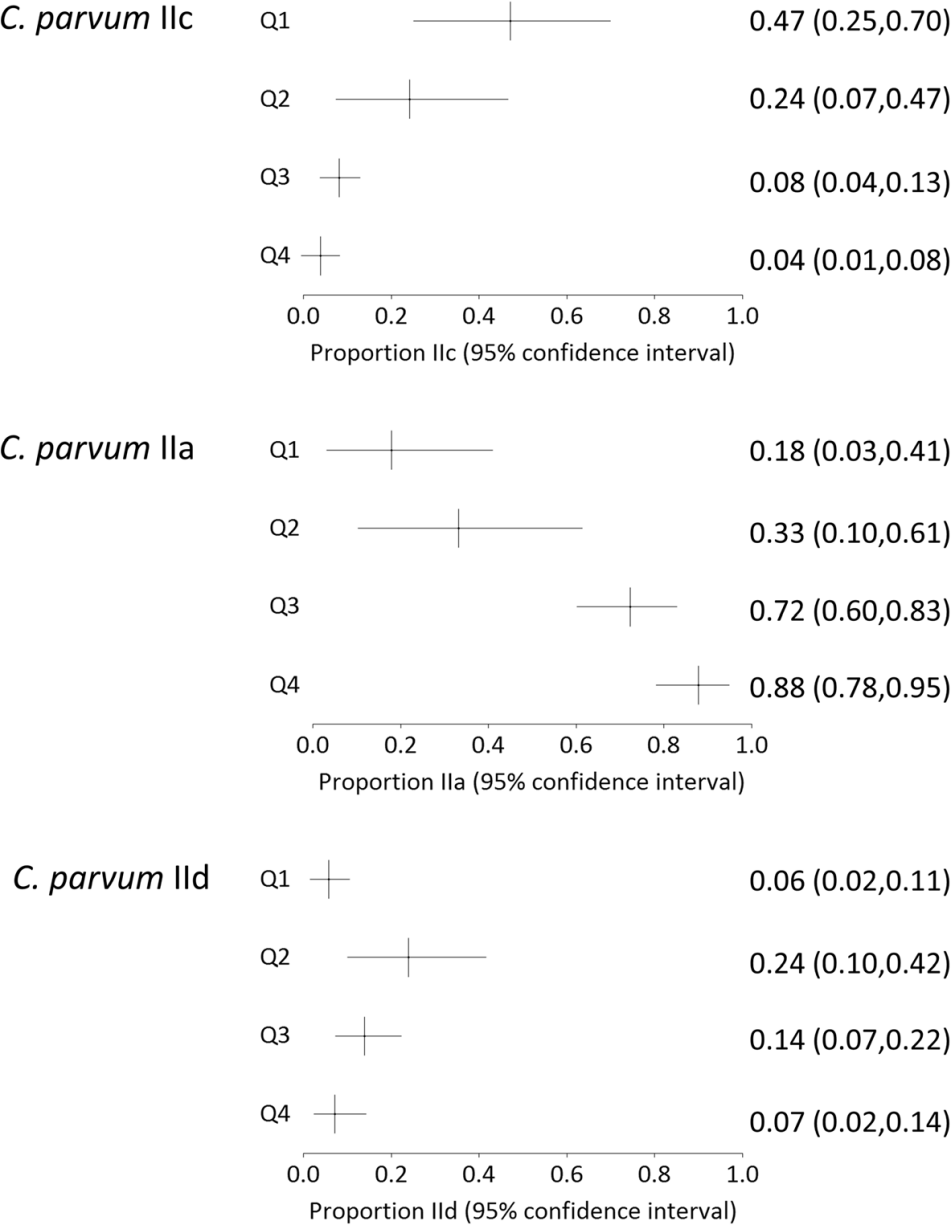
Pooled relative prevalence of three most common *C. parvum* subtypes grouped by quartile of proportion of population with access to improved sanitation with Q1 representing the quartile with least access to improved sanitation

**Table 1:** *Cryptosporidium parvum* subtype frequency distributions in published literature ranked by continent and country.

Reference	Country	Population	Total number *C. parvum* isolates subtyped and numbers of subtypes	Proportion *C. parvum* IIc of *C. parvum* subtyped (%)
Asia				
[48]	Bangladesh	Children	(*n* = 4) (4 × IIm)	0
[49]	Cambodia	Children	(*n* = 4) (4 × IIe )	0
[50]	China	HIV+ patients	(*n* = 2) (2 × IId)	0
[51]	India	Adults and children	(*n* = 14) (6 × IIc; 5 × IId; 3 × IIe)	43
[52]	India	Children	(*n* = 6) (1 × IIc; 1 × IId; 2 × IIm; 2 × IIn)	17
[21]	India	Children	(*n* = 7) (7 × IIc)	100
[53]	India	HIV+ patients	(*n* = 9) (5 × IIb; 3 × IIc; 2 × IIg)	33
[54]	Iran	Children	(*n* = 15) (7 × IIa; 8 × IId)	0
[55]	Iran	Children	(*n* = 17) (6 × IIa; 11 × IId)	0
[56]	Iran	Children	(*n* = 22) (7 × IIa; 15 × IId)	0
[57]	Japan	Humans: not specified	(*n* = 2) (1 × IIa; 1 × IIc)	50
[58]	Japan	Humans: not specified	(*n* = 1) (1 × IIa)	0
[59]	Jordan	Adults and children	(*n* = 2) (1 × IIa; 1 × IId)	0
[60]	Jordan	Children	(*n* = 13) (3 × IIa; 2 × IIc; 8 × IId)	15
[61]	Kuwait	Children	(*n* = 61) (29 × IIa; 12 × IIc; 20 × IId)	20
[9]	Kuwait	Children	(*n* = 59) (27 × IIa; 2 × IIc; 29 × IId;1 × IIf)	3
[62]	Lebanon	Adults and children	(*n* = 5) (5 × IIa)	0
[63]	Malaysia	HIV+ patients	(*n* = 13) (12 × IIa; 1 × IId)	0
[64]	Malaysia	HIV+ patients	(*n* = 1) (1 × IId)	0
[65]	Yemen	Adults and children	(*n* = 7) (7 × IIa)	0
Africa				
[66]	Egypt	Adults and children	(*n* = 5) (5 × IId)	0
[67]	Egypt	Children	(*n* = 14) (7 × IIa; 7 × IId)	0
[17]	Equatorial Guinea	HIV+ patients	(*n* = 10) (7× IIc; 3 × IIe)	70
[68]	Ethiopia	HIV+ patients	(*n* = 82) (71 × IIa; 1 × IIb; 2 × IIc; 5 × IId; 1 × IIe; 2 × If-like 2)	2
[69]	Ethiopia	Adults and children	(*n* = 12) (12 × IIa)	0
[16]	Ghana	Children	(*n* = 37) (30 × IIc; 7 × IIe)	81
[15]	Kenya	Children	(*n* = 19) (19 × IIc)	100
[70]	Madagascar	Children	(*n* = 1) (1 × IIc)	100
[71]	Malawi	Children	(*n* = 2) (1 × IIc; 1 × IIe)	50
[72]	Nigeria	HIV+ patients	(*n* = 1) (1 × IIc)	100
[73]	Nigeria	HIV+ patients	(*n* = 1) (1 × IIc)	100
[74]	Nigeria	Adults and children	(*n* = 1) (1 × IIe)	0
[75]	Nigeria	Children	(*n* = 2) (2 × IIc)	100
[76]	Nigeria	HIV+ Adults	(*n* = 8) (4 × IIc; 4 × unspecified)	50
[18]	Nigeria	Children	(*n* = 23) (2 × IIa; 17 × IIc; 2 × IIi; 2 × IIm)	74
[77]	Sao Tome and Principe	Children	(*n* = 5) (2 × IIa; 3 × IId)	0
[78]	South Africa	Children	(*n* = 5) (1 × IIb; 3 × IIc; 1 × IIe)	60
[79]	South Africa	HIV + children	(*n* = 5) (5 × IIc)	100
[80]	Tunisia	Children	(*n* = 4) (2 × IIa; 2 × IId)	0
[22]	Uganda	Children	(*n* = 15) (10 × IIc; 1 × IIg; 1 × IIh; 3 × IIi 3)	67
Europe				
[81]	Belgium	Adults and children	(*n* = 6) (4 × IIa; 1 × IIc; 1 × IId)	17
[82]	Czech Republic	Adults	(*n* = 1) (1 × IIa)	0
[83]	Denmark	Adults and children	(*n* = 34?) (15 × IIa; Others not stated)	0
[24]	England & Wales, UK	Adults and children	(*n* = 66) (56 × IIa; 1 × IIc; 9 × IId)	2
[84]	Estonia	Human contact with calves	(*n* = 1) (1 × IIa)	0
[85]	France	Adults	(*n* = 1) (1 × IIa)	0
[86]	Ireland	Adults and children	(*n* = 249) (249 × IIa)	0
[87]	Ireland	Adults and children	(*n* = 79) (78 × IIa; 1 × IId)	0
[44]	Italy	AIDS patients	(*n* = 8) (4 × IIa; 4 × IIc)	50
[88]	Portugal	HIV+ patients	(*n* = 25) (9 × IIa; 1 × IIb; 7 × IIc; 8 × IId)	28
[89]	Romania	Children	(*n* = 4) (4 × IId)	0
[90]	Scotland	Adults and children	(*n* = 87) (82 × IIa; 2 × IIc; 2 × IId; 1 × IIg)	2
[91]	Slovak Republic	Humans: not specified	(*n* = 1) (1 × IIa)	0
[92]	Slovenia	Humans: not specified	(*n* = 31) (29 × IIa; 1 × IIc; 1 × IIl)	3
[93]	Spain	Adults and children	(*n* = 12) (7 × IIa; 1 × IIc; 4 × IId)	8
[94]	Spain	Adults and Children	(*n* = 7) (6 × IIa; 1 × IId)	0
[95]	Spain	Children	(*n* = 3) (3 × IIa)	0
[96]	Spain	Adults and children	(*n* = 163) (146 × IIa; 3 × IId; 14 × IIn)	0
[23]	Sweden	Adults and children	(*n* = 108) (89 × IIa; 12 × IIc; 24 × IId; 1 × IIe; 2 × IIo)	11
[25]	The Netherlands	Humans: not specified	(*n* = 13) (9 × IIa; 1 × IIc; 3 × IId)	8
[97]	UK	Humans: not specified	(*n* = 16) (11 × IIa; 1 × IIc; 3 × IId; 1 × IIe)	6
North America				
[98]	Canada	Humans: not specified	(*n* = 7) (7 × IIa)	0
[99]	Canada	Adults and children	(*n* = 5) (5 × IIa)	0
[100]	Canada	Humans: not specified	(*n* = 4) (4 × IIa)	0
[19]	Jamaica	HIV+ patients	(*n* = 7) (7 × IIc)	100
[101]	Mexico	Children	(*n* = 2) (2 × IIa)	0
[102]	USA	Humans: not specified	(*n* = 5) (5 × IIa)	0
[103]	USA	Humans: not specified	(*n* = 30) (30 × IIa)	0
South America				
[104]	Brazil, Argentina	Adults and Children	(*n* = 3) (2 × IIa(mixed with *hominis*); 1 × IIc)	33
[12]	Peru	Children	(*n* = 15) (15 × IIc)	100
[20]	Peru	HIV+ patients	(*n* = 22) (22 × IIc)	100
Australia/Oceania				
[28]	Australia	Humans	(*n* = 14) (14 × IIa)	0
[27]	Australia	Adults and children	(*n* = 21) (21 × IIa)	0
[29]	Australia	Farm workers	(*n* = 7) (5 × IIa; 1 × IId; 1 × IIa/IId)	0
[30]	Australia	Adults and children	(*n* = 80) (70 × IIa 79; 1 × IId)	0
[31]	Australia	Adults and children	(*n* = 49) (48 × IIa; 1 × IId)	0
[26]	Australia	Humans: not specified	(*n* = 32) (30 × IIa; 1 × IIc; 1 × IId)	3
[105]	Australia	Humans: not specified	(*n* = 24) (23 × IIa; 1 × IIc)	4
[32]	Australia	Humans: not specified	(*n* = 4) (4 × IIa)	0
[106]	Australia	Humans: not specified	(*n* = 23) (18 × IIa; 5 × IIc)	22
[33]	New Zealand	Humans: not specified	(*n* = 41) (41 × IIa)	0
Multiple continents				
[47]	Sub-Saharan Africa and Southeast Asia	Children	(*n* = 37) (21 × IIc; 13 × IIe; 3 × IId)	57
[107]	Iran, Malawi, Nigeria, UK, Vietnam	Children	(*n* = 9) (4 × IIa; 2 × IIc; 3 × IId)	22
[108]	Australia + Europe	Adults and children	(*n* = 11) (9 × IIa; 1 × IIc; 1 × IId)	9
[109]	China, Guatemala, India, Kenya, Portugal, Slovenia	Humans: not specified	(*n* = 13) (4 × IIa; 5 × IIb; 4 × IIc)	31

### Study characteristics

Of the 85 studies included, many (*n* = 28) were solely in children, 23 studies included both children and adults, and 14 studies focused on HIV positive or AIDS patients. Some studies did not specify the human population they were studying.

Studies had taken place in a wide variety of countries, including high- and low-income countries and both rural and urban settings.

The number of samples subtyped in most studies was small, ranging from 1 to 249 samples. Included studies were published between 2001 and 2016, with the majority of studies published in the later years, reflecting increased access to molecular techniques.

### *Cryptosporidium parvum* IIc distribution

*Cryptosporidium parvum* IIc was found in 43 studies. In 10 studies, *C. parvum* IIc was the only subtype of *C. parvum* to be found. Across all studies the proportion of *C. parvum* strains typed as IIc using a random effects meta-analysis is 19.0% (95% CI, 12.9–25.9%). However, there was evidence of substantial heterogeneity [Cochran Q = 964.365229, *df* = 84, *P* < 0.0001; I^2^ (inconsistency) = 91.3% (95% CI: 90.1–92.3%)]. The estimates of the pooled proportions for all the GP60 subtypes are shown in Table 2, and Additional file 2: Figure S1 gives the forest plots for the three most common subtypes. It can be seen from Table 2 that the great majority of strains are either IIa, IIc or IId which together account for about 84% of human infections. The remaining 9 subtypes are only detected very rarely, with IIe representing an estimated 2.7% of infections.

**Table 2:** Pooled proportion of GP60 subtypes of *C. parvum*

Subtype	No. of studies^**a**^ (*n* = 85)	Pooled proportion	95% CI
IIa	58	0.53	0.43–0.63
IIb	5	0.0097	0.0058–0.015
IIc	43	0.19	0.13–0.26
IId	36	0.12	0.082–0.16
IIe	11	0.027	0.016–0.039
IIf	2	0.0087	0.0050–0.013
IIg	3	0.0083	0.0048–0.013
IIh	1	0.0075	0.0042–0.012
IIi	3	0.0087	0.0050–0.013
IIm	3	0.0085	0.0049–0.013
IIn	2	0.012	0.0075–0.017
IIo	1	0.0083	0.0048–0.013

^a^Number of studies with at least one of the subtypes

*Abbreviation*: *CI* confidence interval

Even from a simple visual inspection of the data, it was clear that *C. parvum* IIc was particularly common in middle- and low-income countries.

Mbae et al. [15] investigated the distribution of *Cryptosporidium* and diversity of subtypes in children in urban Kenya. Of the 19 samples of *C. parvum* they subtyped, all were the anthroponotic IIc subtype, with no other reported subtypes found. This predominant finding of high numbers of *C. parvum* IIc was also found in children in rural Ghana, with 81% of the subtyped *C. parvum* samples found to be the IIc subtype [16]. This finding was also replicated in HIV-positive patients in Equatorial Guinea [17], children in Nigeria [18], HIV-positive patients in Jamaica [19], both HIV-positive patients and children in Peru [12, 20] and children in both India and Uganda [21, 22].

In contrast, high income countries reported much lower numbers of *C. parvum* IIc as a proportion of total *C. parvum* subtyped. Often European studies did not find *C. parvum* IIc amongst their samples, or if they did it tended to be at low levels. Insulander et al. [23] studied adults and children in Sweden and found a *C. parvum* IIc proportion of just 11%. This finding was replicated by Chalmers et al. [24] studying adults and children in England and Wales who found *C. parvum* IIc in 2% of their *C. parvum*-subtyped samples, and Wielinga et al. [25] who reported a *C. parvum* IIc proportion of 8% from humans in the Netherlands. Studies in Australia and New Zealand tended to report either a very low proportion of *C. parvum* IIc, e.g. Waldron et al. [26] reported a proportion of 3%, or they did not find any *C. parvum* IIc within their samples [27–33].

In order to further investigate the possible drivers of the heterogeneity, we undertook regression analysis of all studies reporting data from a single country where we could allocate GDPpc, sanitation and water coverage data. The results of the analyses are shown in Table 3. It can be seen that in the single predictor analysis that increasing GDP, improved access to sanitation and water supply are all strongly associated with a reduced relative proportion of *C. parvum* IIc. Also, those studies that are focused primarily on people with HIV show a greater relative proportion of *C. parvum* IIc. In the final model both GDP and % access to improved water was dropped from the model leaving % access to improved sanitation, year of publication and whether the study focus was on people living with HIV. The % access to improved sanitation and focus on HIV was particularly strong. The relative proportion of *C. parvum* IIc declined by 3.3% (95% CI: 2.8–4.4%) for every 1% increase in national sanitation coverage. Similarly, the relative proportion of *C. parvum* IIc was 3.4 (95% CI: 1.4–8.2) times greater in studies focusing on people with HIV.

**Table 3:** Negative binomial meta-regression analyses of proportion of *C parvum* that were IIc across 80 analysable studies (studies reporting data from a single country where GDPpc, sanitation and water coverage data could be allocated)

Predictor	Single predictor analyses			Final model		
	Proportion ratio	95% CI	*P-*value	Proportion ratio	95% CI	*P-*value
Log10 GDP per capita US$2005	0.303	0.167–0.549	0.0001			
% population with access to improved sanitation	0.969	0.955–0.983	<0.0001	0.967	0.956–0.972	<0.0001
% population with access to improved drinking water	0.943	0.914–0.975	0.0002			
Year of publication	0.907	0.815–1.022	0.108	0.888	0.794–0.994	0.039
Study focus on HIV-positive individuals	3.263	1.096–9.717	0.034	3.414	1.428–8.162	0.006

*Abbreviation*: *CI* confidence interval

Additional file 2: Figure S1 indicates proportion of *C. parvum* IIc found in studies ordered in increasing sanitation coverage of country of study. Figure 2 shows the pooled relative prevalences for the three most common subtypes by quartile of proportion of the population with access to improved sanitation. *Cryptosporidium parvum* IIc is seen in a higher proportion in countries with low % sanitation coverage, and the proportion of *C. parvum* IIc seen in countries with high % sanitation coverage is much lower, or even none at all. This is in contrast to the subtype IIa, which appears to be seen at a higher proportion in countries with a higher % sanitation coverage, and subtype IId which shows a mixed picture but appears to cluster in Arabic countries. We did not include IIe in this analysis as the numbers were small - IIe was found in small numbers in 11 studies, of which 6 were in the lowest quartile for sanitation provision and one from a mix of countries most of which would have been in the lowest quartile. No IIe strains were reported from countries in the highest quartile.

## Discussion

This is the first systematic study, to our knowledge, investigating worldwide prevalence of *C. parvum* IIc and correlating this with GDP and sanitation data. We have illustrated a striking finding of high proportion of *C. parvum* IIc in low- and middle-income countries and extremely strong relationship between *C. parvum* IIc proportion and GDP and inadequate access to improved sanitation. This is especially pertinent when considering the World Health Organisation Millennium Development Goal of improved sanitation as many low-income countries have made little or no progress towards this goal [34].

The subtype *C. parvum* IIc is interesting as it is different to other *C. parvum* in that it is considered anthroponotic whereas other subtypes of *C. parvum* are zoonotic and tend to infect a wide range of animals in addition to humans. *Cryptosporidium parvum* IIc has never been found in livestock or pet animals, although there are three reports of a particular IIc subtype (IIcA5G3J) being found in hedgehogs [35–37]. However, these may not reflect true infection, rather ingestion from an environment faecally contaminated with *C. parvum* IIc from human waste. In contrast, *C. hominis*, the species of *Cryptosporidium* defined by its predominantly anthroponotic transmission characteristics, has been reported in livestock [7] and also recently in domestic dogs in Spain [38]. This review clearly demonstrates that it is anthroponotic *C. parvum* that is causing the majority of disease in low- and middle-income countries, rather than zoonotic *C. parvum*. This is in spite of the often close proximity people may have with animals in low-income settings, and is thus more likely related to the widespread faecal contamination of both food and water sources in these settings. This finding will have implications for public health and should influence measures to prevent infection and risks of ongoing transmission.

The strong association between *C. parvum* IIc and inadequate access to improved sanitation is worthy of comment. Given the prior association between outbreaks of cryptosporidiosis and waterborne disease [39], one would have expected the association to be strongest with inadequate access to drinking water. The fact that this is not the case needs to be explained. It is accepted that *C. hominis* is more common in low-income countries and that *C. hominis* is a human pathogen [40] (although it has rarely been reported in livestock [7] and in dogs [38]). Epidemiological studies that have found inadequate household sanitation is a risk factor for cryptosporidiosis infection in low-income countries including India [41], Venezuela [42] and Peru [43]. Therefore, the higher prevalence of *C. hominis* compared to *C. parvum* in low income countries observed suggests that anthroponotic transmission rather than zoonotic transmission is the main pathway in such countries, and this may explain the higher relative proportion of *C. parvum* IIc in those same countries. However, there may be other reasons for seeing a higher relative proportion of *C. parvum* IIc, including increased virulence or prolonged shedding for example. Our study focused on *C. parvum* subtypes, and thus *C. hominis* subtypes were not included. It is possible that certain *C. hominis* subtypes would also show increased prevalence in lower income countries, particularly for example IbA10G2 which is thought to be more virulent [12], but this would need to be systematically studied in order to make any conclusions.

Data within this systematic review were not robust enough to draw conclusions about the virulence of *C. parvum* IIc. However, it is possible that *C. parvum* IIc is more virulent than other *C. parvum* subtypes, as we know now *C. parvum* IIc is causing the majority of *C. parvum* infections in low-income settings where morbidity and mortality due to *Cryptosporidium* infections is highest. However, our data cannot provide the evidence for this, and it is plausible that there are host susceptibility factors involved which make *C. parvum* IIc more prevalent, but not necessarily more virulent, as there may be other subtypes that may cause less cases, but potentially more virulent disease. In addition, as *C. parvum* IIc is anthroponotic it may act more like *C. hominis* than zoonotic *C. parvum* and research has previously suggested that *C. hominis* is more virulent than *C. parvum* [11]. One observational study [44] stratified AIDS patients with cryptosporidiosis into three groups (mild, moderate and severe) based on symptom features, and in the severe group the only subtype of *Cryptosporidium* found was *C. parvum* IIc. In addition, they suggested that wasting syndrome was strongly linked to the subtype IIc, with wasting seen in four out of four patients with IIc subtype, whereas no wasting syndrome was seen in patients with Ia (1 patient) and IIa (4 patients) subtypes of *Cryptosporidium*. The study was, however, limited by its small size as it included only nine cases of cryptosporidiosis, and the patient population included AIDS patients only. It does though, raise the question of whether *C. parvum* IIc causes more severe disease demonstrating the need for further research to address this. Well conducted, adequately powered epidemiological studies investigating differences in clinical symptoms, illness duration and long-term sequelae between different subtypes would be able to provide data to answer this question.

There are several limitations of this review. The first is that it relied on published studies to report the proportion of *C. parvum* IIc, and these studies are not systematic studies of subtyping. Instead subtyping tends to be done on a subset of samples, often due to expense, which can mean small numbers of subtyped samples are available for analysis. In addition, earlier studies often did not subtype, before *gp60* subtyping became more widespread, and thus these studies could not be included in this review. *Gp60* subtyping is the commonly used method for subtyping *Cryptosporidium*, but concerns have been raised as to whether it could be missing some genetic diversity and the role of multi-locus typing has been investigated [45]. However, a consensus is yet to be reached and as such *gp60* subtyping has remained the mainstay of *Cryptosporidium* subtyping, although recently a working group has been set-up to implement and establish a multi-locus genotyping scheme for *Cryptosporidium* [46]. Regarding the statistical analysis relating to GDP and percentage access to improved sanitation and improved water supply, multiple variate parameters were not undertaken due to high degrees of co-lineality.

With an increased emphasis on *Cryptosporidium* as a pathogen capable of causing severe disease and now recognised to be the second leading cause of death from diarrhoeal disease in children under the age of five [5], this study emphasises how anthroponotic *C. parvum* IIc is disproportionately affecting low income countries and demonstrates a clear link with sanitation.

There is an estimated disease burden of 7.6 million diarrhoeal cases due to *Cryptosporidium* annually [47] and recent understanding has highlighted the importance of anthroponotic *Cryptosporidium* in causing these infections in sub-Saharan Africa and South Asia, with *C. hominis* responsible for 77.8% and *C. parvum* 9.9% of these *Cryptospordium* infections. Of the *C. parvum* cases, 91.9% were anthroponotic, of which the far majority were IIc (57%) and then IIe (35%) [47]. Extrapolating this information suggests a *C. parvum* disease burden of three quarters of a million cases, with more than half of these caused by IIc, resulting in significant morbidity and mortality from the parasites comprising this GP60 subtype. This corresponds to our study finding of high proportion of *C. parvum* IIc in low-income countries, where the biggest burden of diarrhoeal disease in children is seen, and clearly any intervention to reduce this is desirable.

Given the disease burden associated with cryptosporidiosis in low-income countries and the current lack of an effective treatment or vaccine, there is a need for improved prevention. Our findings and those of the few other studies that have investigated suggest that improving sanitation provision may be the most important intervention to reduce the burden of disease from cryptosporidiosis and its associated increased risk of death in young children. We would support the importance of achieving the Sustainable Development Goal on sanitation provision.

## Conclusions

Our systematic study has shown that anthroponotic *C. parvum* IIc predominates in lower-income countries with poor sanitation and in HIV positive individuals, in contrast to higher-income countries where it is rarely evident. Considering the large disease burden of cryptosporidiosis in low-income countries and the post-infectious virulence of other anthroponotic *Cryptosporidium* species such as *C. hominis*, *C. parvum* IIc plays an increasingly apparent role in this disease process. Given the current lack of effective treatment or vaccine, interventions to improve sanitation provision may be the best option to try and reduce the cryptosporidiosis disease burden and associated childhood deaths in lower income countries.

## Additional files

Additional file 1: **Table S1.** PRISMA checklist. (DOC 62 kb)

Additional file 2: **Figure S1.** Forest plots ordered by increasing sanitation coverage in country of study for *C. parvum* IIc (a) *C. parvum* IIa (b) and *C. parvum* IId (c) illustrating the increased proportion of *C. parvum* IIc found in countries with poor sanitation coverage and low proportion of *C. parvum* IIc in countries with high % sanitation coverage, in comparison to *C. parvum* IIa which is frequently seen in a higher proportion in countries with high % sanitation coverage and *C. parvum* IId which appears to cluster in Arabic countries. Vertical line within the figures equals the pooled relative proportion of all studies. (PDF 1131 kb)
