# Supplementary material for: Anthroponotic transmission of Cryptosporidium parvum predominates in countries with poorer sanitation: a systematic review and meta-analysis
Source: Parasit Vectors. 2019 Jan 8;12:16. doi: 10.1186/s13071-018-3263-0 (PMC6323761; doi:10.1186/s13071-018-3263-0)

Supplementary figure 1. Forest plots ordered by increasing sanitation coverage in country of study for a) *C. parvum* IIc b) *C. parvum* IIa and c) *C. parvum* IId illustrating the increased proportion of *C. parvum* IIc found in countries with poor sanitation coverage and low proportion of *C. parvum* IIc in countries with high % sanitation coverage, in comparison to *C. parvum* IIa which is frequently seen in a higher proportion in countries with high % sanitation coverage and *C. parvum* IId which appears to cluster in Arabic countries. Vertical line within the figures equals the pooled relative proportion of all studies.

Figure S1a. *C parvum* IIc

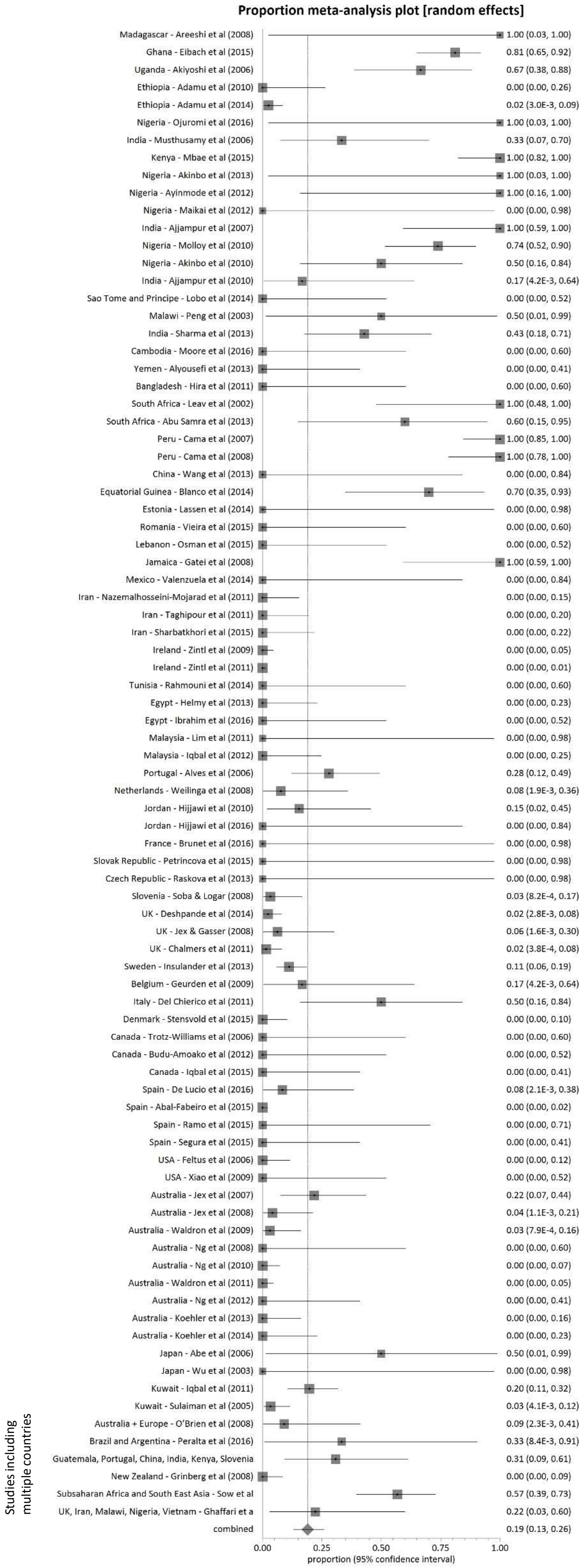

Figure S1b. *C parvum* IIa

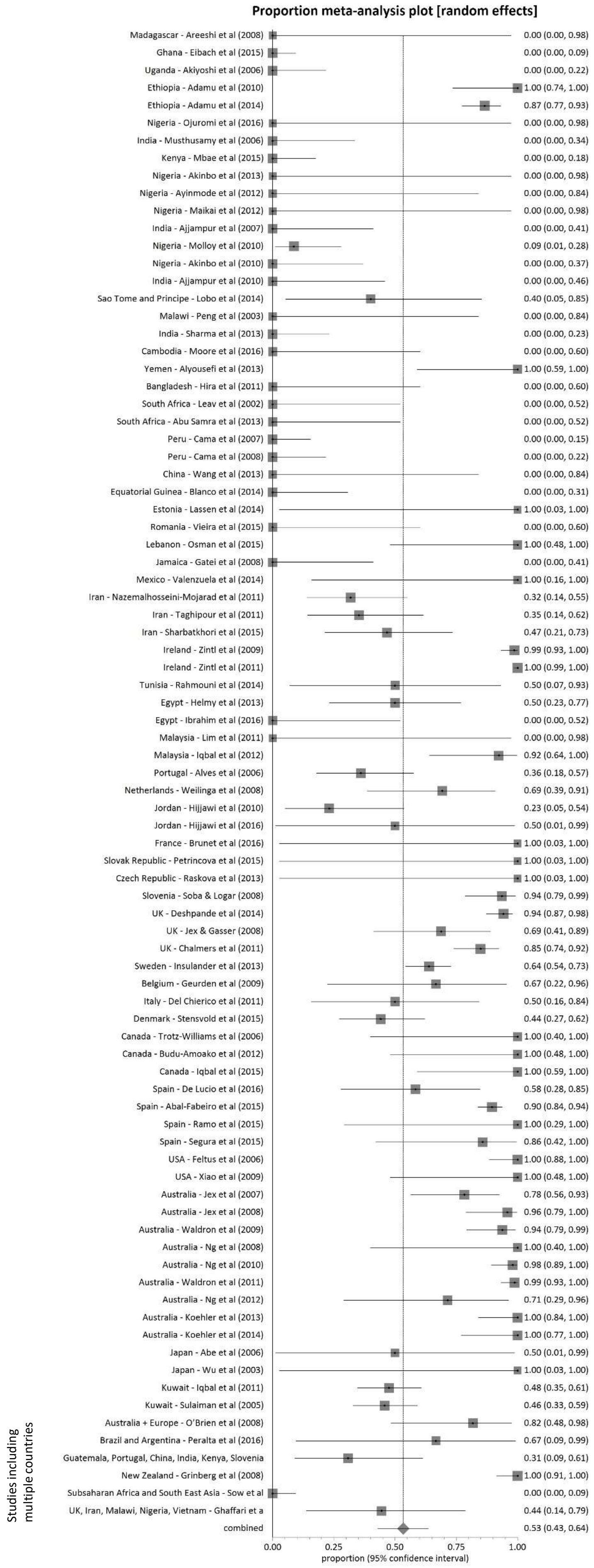

Figure S1c. *C parvum* IId

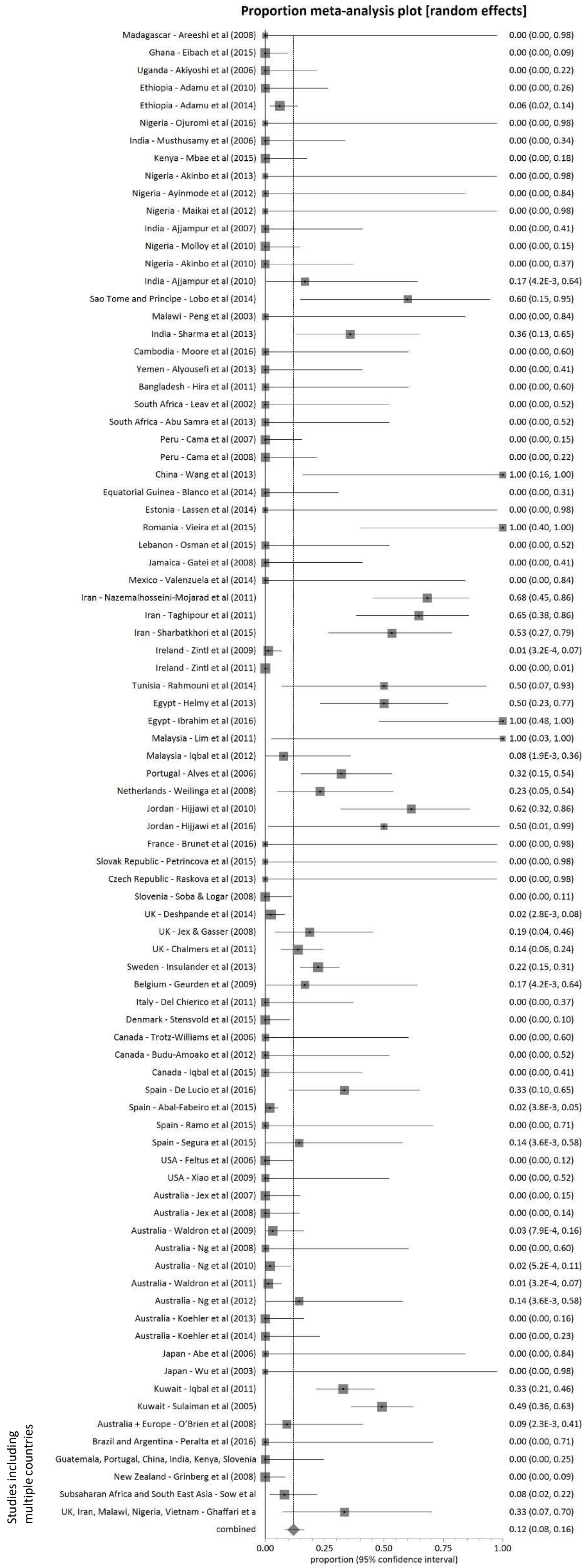

Supplement: Supplementary file 2 — Figure S1. Forest plots ordered by increasing sanitation coverage in country of study for C. parvum IIc (a) C. parvum IIa (b) and C. parvum IId (c) illustrating the increased proportion of C. parvum IIc found in countries with poor sanitation coverage and low proportion of C. parvum IIc in countries with high % sanitation coverage, in comparison to C. parvum IIa which is frequently seen in a higher proportion in countries with high % sanitation coverage and C. parvum IId which appears to cluster in Arabic countries. Vertical line within the figures equals the pooled relative proportion of all studies. (PDF 1131 kb) [file 13071_2018_3263_MOESM2_ESM.pdf]
